# Occupational Radiation Exposure to the Extremities of Medical Staff during Hysterosalpingography and Radionuclide Bone Scan Procedures in Several Nigerian Hospitals

**DOI:** 10.4274/mirt.96158

**Published:** 2016-09-29

**Authors:** Nnamdi Norbert Jibiri, Tawakalitu Oluwatoyin Akintunde, Musa Yusuf Dambele, Christopher Jimoh Olowookere

**Affiliations:** 1 University of Ibadan, Radiation and Health Physics Research Laboratory, Department of Physics, Ibadan, Nigeria; 2 University of Ibadan, University College Hospital, Clinic of Nuclear Medicine, Ibadan, Nigeria; 3 Ajayi Crowther University, Faculty of Natural Sciences, Department of Physics, Oyo Town, Nigeria

**Keywords:** Radiation dose, hysterosalpingography, bone scan, thermo-luminescent dosimeter, hand dose

## Abstract

**Objective:**

The practice of regular dose measurement helps to ascertain the level of occupational dose delivered to the staff involved in diagnostic procedures. This study was carried out to evaluate the dose exposed to the hands of radiologists and a radiologic technologist carrying out HSG and radionuclide bone scan examinations in several hospitals in Nigeria.

**Methods:**

Radiation doses exposed to the hands of radiologists and a technician carrying out hysterosalpingography (HSG) and bone scan procedures were measured using calibrated thermo-luminescent dosimeters. Five radiologists and a radiologic technologist were included in the study for dose measurement.

**Results:**

The study indicates that each radiologist carried out approximately 2 examinations per week with the mean dose ranging between 0.49-0.62 mSv per week, resulting in an annual dose of 191 mSv. Similarly, the occupational dose delivered to both the left and right hands of a radiologic technologist administering ^99m^Tc-methylene diphosphonate (MDP) without cannula and with cannula were 10.68 (720.2) and 13.82 (556.4) mSv per week (and per annum), respectively. It was determined that the left hand of the personnel received higher doses than their right hand.

**Conclusion:**

The estimated annual dose during HSG is far below the annual dose limit for deterministic effects, however, it is greater than 10% of the applicable annual dose limit. Hence, routine monitoring is required to ensure adequate protection of the personnel. The total annual dose received during the bone scan exceeds the annual dose limit for both hands, and the dose to either left or right hand is greater than the dose limit of 500 mSv/yr. The radiologists monitored are not expected to incur any deterministic effects during HSG examinations, however, accumulated doses arising from the scattered radiation to the eyes, legs, and neck could be substantial and might lead to certain effects. More staff are required to administer 99mTc-MDP in Nigerian institutions to prevent excessive doses to personnel.

## Abstract (tr)

**Amaç:**

Düzenli doz ölçümü uygulaması tanı yöntemlerinde yer alan personele verilen mesleki dozun düzeyini tespit etmek için yardımcı olur. Bu çalışma Nijerya’da çeşitli hastanelerde HSG ve radyonüklid kemik sintigrafisi incelemelerini yürüten bir radyoloji teknisyeni ve radyologların ellerinin maruz kaldığı dozu değerlendirmek amacıyla yapılmıştır.

**Yöntem:**

Histerosalpingografi (HSG) ve radyonüklid kemik sintigrafisi incelemelerini yürüten bir radyoloji teknisyeni ve radyologların ellerinin maruz kaldığı radyasyon dozu kalibre edilmiş termo-lüminesan dozimetreler kullanılarak ölçüldü. Beş radyolog ve bir radyoloji teknisyeni doz ölçümü için çalışmaya dahil edildi.

**Bulgular:**

Bu çalışma her radyoloğun haftada 2 inceleme yaparak ortalama haftalık dozun 0,49-0,62 mSv arasında değiştiğini, yıllık 191 mSv dozla sonuçlandığını göstermiştir. Benzer şekilde, radyoloji teknisyeninin sol ve sağ ellerine sırasıyla kanülsüz ve kanüllü ^99m^Tc-metilen difosfonat (MDP) uygulamasında verilen haftalık (ve yıllık) dozlar 10,68 (720,2) ve 13,82 (556,4) mSv olarak bulunmuştur. Personelin sol elinde sağ elden daha yüksek doz aldıkları tespit edilmiştir.

**Sonuç:**

HSG sırasında tahmini yıllık doz deterministik etkiler için bildirilen yıllık doz sınırının çok altındadır, ancak uygulanabilir yıllık doz sınırının %10’undan daha fazladır. Bu nedenle, personelin yeterli korunmasını sağlamak için rutin izleme gereklidir. Kemik taraması sırasında alınan toplam yıllık doz her iki el için yıllık doz sınırını aşmaktadır ve sol veya sağ el dozu 500 mSv/yıl doz sınırından daha fazladır. İzlenen radyologların HSG muayenelerine bağlı herhangi bir deterministik etkiye uğraması beklenmemektedir, ancak gözler, bacaklar ve boyuna saçılan radyasyondan kaynaklanan birikmiş dozlar önemli olabilir ve belirli etkilere yol açabilir. Personele aşırı dozu önlemek için Nijerya kurumlarında 99mTc -MDP uygulayabilecek daha fazla insan gücü gereklidir.

## INTRODUCTION

The use of both ionizing and non-ionizing radiations for medical imaging and treatment is rapidly increasing. The use of these medical tools has led to several breakthroughs in both diagnosis and treatment. The rapid development of imaging technology has contributed largely to progress of simple and complex diagnostic procedures as well as interventional radiology (1). Hysterosalpingography (HSG) is a diagnostic procedure performed to determine if the fallopian tubes are patent (open), and to see if the structure and size of the uterine cavity are normal. This is a non-invasive procedure usually performed after the menstrual period has ended to prevent interference with an early pregnancy. It is performed by positioning a woman under a fluoroscope (real-time imager) on a table. The gynecologist or radiologist examines the patient and fills the uterus with contrast medium in order to visualize the outline of the inner size and shape of the uterus and fallopian tubes clearly. X-ray images are obtained during the introduction of the contrast medium using a tube. During the filling of the uterus with the contrast medium, the fingers and the lower extremities of the gynecologist or radiologist is exposed to radiation (2,3).

The HSG is a common procedure carried out in Nigeria essentially in infertile women. This is because of the cultural practice of disparaging women with infertility problems. The HSG procedure is also used a few months after a tubal sterilization procedure to make sure that the fallopian tube has not been completely blocked. The common indication for the use of HSG in Nigeria is infertility. Other indications include, but are not limited to, the evaluation of: pelvic pain, irregular vaginal bleeding, congenital abnormalities or anatomic variants (4). Other alternative procedures to HSG are laparoscopy, sonohysterosalpingogram and hysteroscopy. Nevertheless, HSG remains the most commonly performed procedure to evaluate tubal patency (2).

HSG procedure requires the radiologist or gynecologist (who is not trained to handle ionizing radiation) to hold the cannula and inject the contrast medium into the cervix of the patient while she is being irradiated. The supporting personnel also remain close to the patient. Though, a lead apron is worn, the hands of the radiologist are covered in latex gloves, making their hands vulnerable to x-rays. The exposure of the radiologists’ hands to x-rays during this procedure is a continuous and inevitable experience during HSG procedures, hence the need to evaluate the dose exposed to the hands.

The use of ^99m^Tc has gained wide acceptance in nuclear medicine practice due to its advantages related to its specific characteristics. The physical half-life of ^99m^Tc used in radionuclide bone scan is 6.02 hours (5,6), thus it exposes a fairly low dose per unit intake due to its short half-life and radiation spectrum (7). It has an energy of 140 keV that is sufficient enough to be detected by the gamma camera through the body. The reason for the choice of ^99m^Tc in bone scintigraphy arose because of its characteristic qualities. Bone scintigraphy/scan is one of the most common applications of ionizing radiation in nuclear medicine. Radionuclide bone scan is a diagnostic procedure used to evaluate the distribution of active bone formation in the body. The radiopharmaceutical is injected intravenously with and without cannula to the patient by a radiologic technologist and is distributed via blood flow throughout the body. It therefore passively diffuses into the extravascular and extracellular spaces, and bind to hydration shell around the bone crystal (8). The use of ^99m^Tc in patients undergoing bone scan procedure presents special concerns for the assessment of radiation dose (9) and the attendant risk to the administering staff. As a result of the need for radiation protection of the administering staff, doses exposed to the hands of the radiologic technologist were measured.

More than 92% of all investigations carried out at the Department of Nuclear Medicine, University College Hospital (UCH), Ibadan, is bone scan using ^99m^Tc. The aim of this present study was to evaluate the radiation doses exposed to the hands of radiologists and a radiologic technologist carrying out HSG and radionuclide bone scan procedure in some selected hospitals in two major cities in Nigeria. The results obtained were compared with the recommended dose limits.

## MATERIALS AND METHODS

The study involving HSG was carried out between February and May 2013 in five selected hospitals in the Mega City of Lagos, while the bone scan study was carried out at the UCH (a teaching hospital), Ibadan. The selection of the study centers in Lagos was based on the availability of facilities for carrying out the HSG procedure at each hospital. In addition, the facilities of the hospitals have been previously monitored in compliance with the Nigerian Nuclear Regulatory Authority (a body responsible for regulation of nuclear activities in Nigeria) policy. Forty-eight patients and five radiologists participated in the HSG study. The goals of the researchers were discussed with the patients and the staff at the beginning of the study. Five private health care centers included in the study are; Rays and Waves Radiological Centre, Ifako (RWRC); Fanic Diagnostic Centre, Dopemu (FDC); Precise Medical Diagnostix, Iju (PMD); Royal Hospital, Ojodu (RHH); and The Shield Hospital, Eko (TSH). Fifty-two patients who have been injected during the HSG procedure carried out within the study period were included in the study. The doses received by the radiologic technologist during the bone scan procedures using ^99m^Tc-methylene diphosphonate (MDP) were monitored at the Nuclear Medicine Department of UCH, Ibadan. The radiologic technologist who carried out the investigation was permitted to undertake the examination as part of his routine work by the law of UCH. Thermo-luminescent dosimeters (TLD) pellets were attached to his fingers. The HSG procedures were carried out by certified radiologists who were by the law of our country permitted to examine patients as part of their daily duty, and the study was also carried out on the radiologists themselves.

### Radiation Dose Measurement during Hysterosalpingography

In order to assess doses exposed to the hands, calibrated TLD pellets obtained from the Lagos State University Radiation Monitoring Service (LASURMS) (a university-based radiation monitoring service provider) were attached to the fingers of both right and left hands of each radiologist prior to the exposure of the patient to radiation. Patients were irradiated with x-ray beam energy ranging between 90 kVp and 99 kVp, and with the tube load in the range of 40-50 mAs depending on patient size. After the first exposure, the used film was removed and a new one was introduced to take the left and right antero-posterior views. This implies that three images were taken while the contrast medium was administered. Each procedure took between 10 and 30 minutes when the patient cooperated fully with the radiologist carrying out the investigation. In certain cases, patients were allowed to rest while the film was being processed and assessed to ensure that the film provided the required diagnostic information. After irradiation, the TLD chips were removed and kept outside the x-ray room to prevent further exposure from scattered radiation. The irradiated dosimeters were calculated by using the RADOS TLD READER RE 2000 of LASURMS.

### Radiation Dose Measurement during Whole Body Bone Scan

Thermo-luminescent dosimeter (LiF-TLDs) pellets with dimension of 4.5 mm (diameter) by 0.9 mm (thickness) were used in the study to determine the dose delivered to the base of the index fingers of the radiologic technologist handling the 99mTc-MDP. TLD pellets with variation of ±5% were selected for the study. The labeled pellets were inserted in a plastic holder that could be adjusted for any size which was worn by the radiologic technologist at the base of index fingers of both hands (left and right). The choice of the index finger was performed according to the convenience of the radiologic technologist and the proximity of the source to the fingers while administering the 99mTc-MDP. A total of 104 TLD pellets were used during the administration of ^99m^Tc-MDP and were evaluated later. The TLD pellets were kept outside the radiation area when not in use. Control TLD pellets were also kept outside the radiation area for measuring the background response of the TLD. The background readings were subtracted from the measured doses. TLD reader (Harsaw 3500) obtained from National Institute for Radiation Protection and Research, Department of Physics, University of Ibadan was used to read the TLD pellets.

## RESULTS

### Result of Finger Doses during Hysterosalpingography

Doses to the patient were measured in terms of personal dose equivalent horsepower (0.07). This is equivalent to the dose in the tissue (soft) below a specified point on the body at a depth of 0.07 mm.

Ten patients were examined by a radiologist in each of the following study centers: RWRC, FDC, PMD and RHH; while eight patients were included in the study carried out in TSH. In each of the centers, one radiologist took part in the study during the period of sixteen (16) weeks. Radiologists were assigned code numbers: R1 (RWRC), R2 (FDC), R3 (PMD), R4 (RHH), and R5 (TSH). Radiologists R1-R4 performed an average of 1.6 HSG procedures per week (≈2) while R5 performed 2 HSG procedures per week.

Table 1 shows the radiation doses (mSv) delivered to the hands of the five radiologists studied during the period of 16 weeks. The range of mean doses to both hands of the radiologists were R1: 0.14-0.31 mSv, R2: 0.21-0.44 mSv, R3: 0.23-0.48 mSv, R4: 0.16-0.36 mSv, and R5: 0.17-0.43 mSv. The radiologist coded R5 carried out eight examinations during the study period. The highest dose during the study was received by the left hand of radiologist R3. A dose close to the maximum value was received by the left hand of radiologist R2. To a large extent, the doses to the left hands of the radiologists were higher than the doses to their right hands. Relatively lower doses were received by R1, R4 and R5 while examining patients P5, P6, and P8.

Table 2 shows the doses accumulated during 16 weeks, estimated annual dose to both hands, mean doses received on each hand during the 16 weeks, and mean dose accumulated in 16 weeks by both hands. The dose range that was accumulated by both hands was 4.86-6.13 mSv, and the corresponding estimated annual accumulated dose ranged between 15.8-19.9 mSv. Among the five radiologist investigated, R1 received the smallest accumulated annual dose of 15.8 mSv.

### Results of Doses Measured during Bone Scan

Tables 3 and Table 4 display the estimated activities of injected radiopharmaceuticals and their administration time. The mean injected activities with and without cannula were 19.6±2.16 mCi and 19.9±1.49 mCi, respectively. The corresponding mean administration times were 33.3±9.4 seconds, and 44.7±8.57 seconds for administration without and with a cannula, respectively.

Table 5 shows the results of the total dose delivered with cannula and without cannula to the right and left hands. The mean doses to the right and left hand (without cannula) were 0.20±0.14 and 0.21±0.17 mSv, respectively. Also the mean doses delivered to the left and right hands were 0.25±0.12 and 0.28±0.15 mSv, respectively. Table 6 demonstrates the estimated daily, weekly and annual doses received during the administration of ^99m^Tc-MDP. The mean doses per day (with cannula) were 1.46±0.56 and 1.31±0.34 mSv for the left and right hand, respectively. Furthermore, the mean doses received per day (without cannula) by the radiologic technologist to the left and right hands were 1.09±0.69 mSv and 1.05±0.64 mSv, respectively.

## DISCUSSION

Regular monitoring of radiation doses received by the extremities of radiologists, physicians and technologists involved in HSG is very important in order to ascertain the level of exposure of the unprotected part of the hands of the personnel who carry out the procedure. The regular dose assessment of hands, eyes, gonads, and legs is essential to ensure that occupational doses received by radiologists are within the recommended annual dose limit. The results of this study demonstrated that each radiologist performed an average of 2 HSG procedures per week. The two patients per week schedule on a regular basis amounts to 154 HSG examinations per annum. A mean dose of 0.62 mSv received per examination (maximum mean dose per exam from both hands) yields a total dose of 191 mSv per annum. This is relatively higher than 3/10^th^ (150 mSv) and about 141 mSv higher than 1/10th (50 mSv) of the limit. The annual dose limit for deterministic effects to the hands and legs as well as the skin is set to 500 mSv averaged over 1 cm^2^ area of skin regardless of the exposed field. Routine dose monitoring is legally required if 1/10^th^ of the limit is reached (10). An estimated annual dose for the radiologists monitored during this study was calculated by multiplying the mean dose (upper limit) obtained for an HSG procedure by the estimated workload (number of procedures) for one year. Since the annual dose exceeds 1/10^th^ of the limit in the present study, it is essential to routinely monitor the radiation doses received by radiologists.

Although, the annual estimated dose obtained from the workload (HSG) is greater than 3/10^th^ of the limit, it is not unlikely that the radiologists also perform other procedures such as interventional radiology and cardiac radiological procedures which involve long fluoroscopic times, hence leading to higher doses (11,12). Besides, doses to the legs and lens of the eyes were not measured. Measurement of these values might significantly increase the annual dose.

Table 1 shows that relatively higher doses were delivered to the left hands of most of the radiologists. The doses to the left hand for all radiologists ranged from 0.15-0.63 mSv while the range of doses to the right hand was 0.13-0.34 mSv. The few exceptions to this trend are R1 (P7) and R4 (P8), while the results of R5 (P4) were comparable. The reason for higher doses could be attributed to the irradiation of the left hand that is used to hold the cannula in place and to prevent slipping off. This could also be attributed to most radiologist’s leaning their left hands towards the cathode of the x-ray tubes, thus increasing received doses due to heel effects. The reported doses to the left hand in this study are in agreement with an earlier study, which reported that (interventional) physicians received higher doses on their left wrist during interventional radiology and cardiology procedures (13). It is evident from Table 1 that slight variations exist among received doses by radiologists. The variation could be attributed to various factors such as patient’s body size (14), skill and the experience of the physicians (15), and the exposure parameter selected (16) during examinations.

Using the accumulated dose during the study period as seen in Table 2, the calculated accumulated annual doses indicate a range of 15.8-19.9 mSv, which implies that it is still within the recommended limit of 500 mSv/yr. The occupational doses received by radiologists in this study are below the acceptable dose limit and therefore, does not constitute serious health hazards if doses to other extremities are not considered. However, the calculated occupational dose based on the typical workload of a radiologist indicates that routine dose monitoring is required in Nigerian hospitals for effective radiation protection of radiology staff.

With regards to radionuclide bone scan procedures, a comparison of Table 3 and Table 4 show that the exposure time of the procedures carried out with a cannula is longer than the duration of administration without one. This excess mean time of 11.4 seconds between the two modes of administering the radiopharmaceutical could be due to the difficulty in applying the cannula. The extra time spent in the administration might have led to the apparently higher dose received by the radiologic technologist using the cannula, which is evident in Table 5. Apparently, the mean dose received by radiologic technologists is higher when a cannula was used to administer the ^99m^Tc-MDP. Table 5 indicates that the dose delivered to the left hand was higher than the dose delivered to the right hand of the staff by a factor of 1.04 without a cannula, and was higher by a factor of 1.12 when a cannula was used. In addition, the total dose received while using a cannula is higher than the total dose received when a cannula was not used by the administering staff by a factor of 1.30.

The estimated total doses per annum received by a staff administering ^99m^Tc-MDP with and without a cannula were 720.2 mSv and 556.4 mSv, respectively. We assumed that each radiologic technologist worked five days per week, and that he was available to administer the radiopharmaceutical throughout 52 weeks of the year. The total doses received by the radiologic technologist without and with a cannula were 10.68 mSv and 13.82 mSv, respectively. Based on the assumption that the staff does not go on vacation but rests only on weekends, the total annual dose that could be delivered to the staff would be 1276.6 mSv. The estimated occupational dose per annum exceeds the annual limit of 500 mSv to the extremities, hands and feet, and skin (17,18) by a factor of 2.6. The relatively higher dose recorded in this study could be related to the assumption that the radiologic technologist handling ^99m^Tc-MDP carries out the administration throughout the whole year as opposed to working on a rotating schedule as has been earlier reported by Pant et al. (17). Moreover, these results are the upper limit of the estimated dose data. The trend found in the present study calls for the engagement of more technical staff who could administer the radiopharmaceuticals in turn or on rotation basis, which in turn will help reduce the dose received by an individual staff per year.

The total dose delivered to the left and right hands were 663.0 and 613.6 mSv, respectively, with a mean dose of 638.3 mSv to each hand. The mean total dose to each hand exceeds the annual dose limit of 500 mSv.

## CONCLUSION

The radiation doses delivered to the hands of the radiologists who carry out HSG procedures were measured in five hospitals in Nigeria (within Lagos). Mostly, higher doses were delivered to their left hands. The estimated annual dose calculated according to the workload revealed that the recommended annual limit for the extremities are not surpassed. Nevertheless, the estimated annual dose exceeds 10 percent of the annual dose to the extremities, thus indicating the need for monitoring individual doses to the extremities of the radiologists.

However, the estimated annual dose received by a staff administering the 99mTc-MDP during bone scan exceeded the annual dose limit for health workers. The high dose recorded during the bone scan indicates the need for involvement of additional technical staff in the injection of ^99m^Tc-MDP so as to reduce the dose received by the few radiologic technologists involved in the administration of the radionuclide.

### Acknowledgements

The authors would like to thank all the participating radiologists, radiologic technologists, and the managements of the included hospitals in Lagos. The staff of Nuclear Medicine Department, UCH Ibadan are gratefully acknowledged. Our sincere thanks go to the management and staff of Lagos State University Radiation Monitoring Service (LASURMS) for providing TLD pellets and Reader for dose assessment. Our sincere appreciations also go the staff of National Institute for Radiation Protection and Research, University of Ibadan for enabling their TLD Reader and TLD pellets.

### Ethics

Ethics Committee Approval: The radiologic technologist who carried out the investigation was permitted to undertake the examination as part of his routine work by the law of University College Hospital. Thermo-luminescent dosimeters pellets were attached to his fingers. The hysterosalpingography procedures were carried out by certified radiologists who were by the law of our country permitted to examine patients as part of their daily duty, and the study was also carried out on the radiologists themselves, Informed Consent: Consent form was filled out by all participants.

Peer-review: External and internal peer-reviewed.

Financial Disclosure: No financial support/Grant was received in carrying out the study.

## Figures and Tables

**Table 1:**
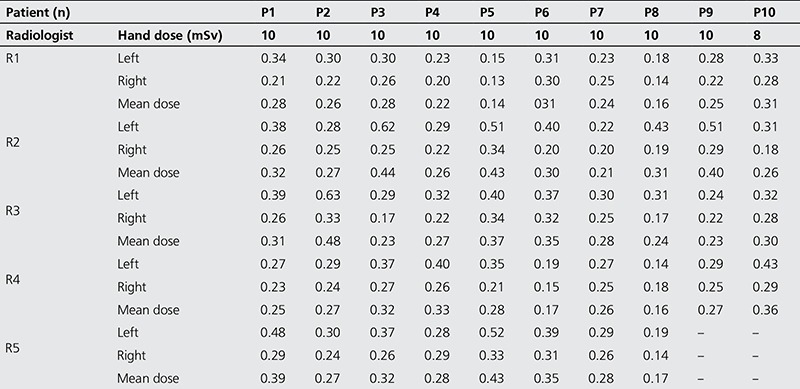
Distribution of occupational doses among different radiologists

**Table 2:**
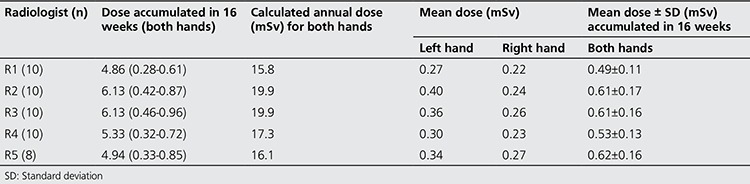
Mean, accumulated and calculated annual occupational dose

**Table 3:**
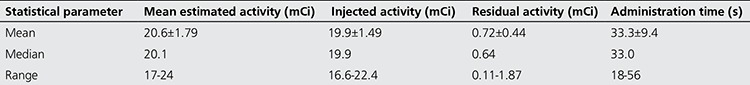
Activity of radiopharmaceuticals administered to patients (no cannula)

**Table 4:**
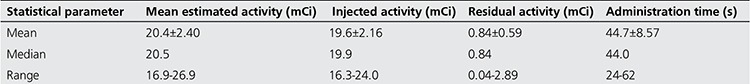
Activity of radiopharmaceuticals administered to patients (with cannula)

**Table 5:**
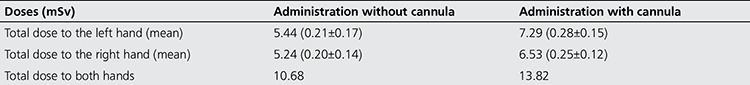
Total dose to each hand of the radiologic technologist (with cannula or without cannula)

**Table 6:**
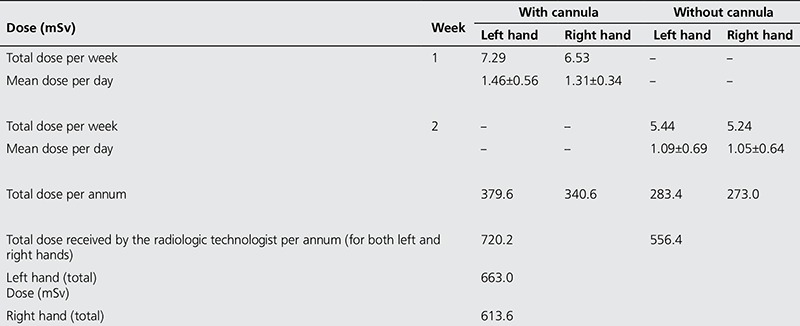
Estimated daily, weekly and annual doses received by administering radiologic technologist
